# Bilateral Thalamic Infarction Secondary to Deep Cerebral Venous Thrombosis: A Diagnostic Challenge Associated With Hormonal Therapy

**DOI:** 10.7759/cureus.109386

**Published:** 2026-05-21

**Authors:** Maria Fernanda Mariscal Reyes, Jorge David Villanueva Cuevas, Sara Monserrat Rosas Noriega, Zarú Isaac Urrutia López, Maria Del Rayo Mendez Palacios

**Affiliations:** 1 Medicine, Benemérita Universidad Autónoma de Puebla, Puebla, MEX; 2 Internal Medicine, Hospital Regional de Alta Especialidad del Instituto de Seguridad y Servicios Sociales de los Trabajadores del Estado (ISSSTE) Puebla, Puebla, MEX; 3 Internal Medicine, Hospital General de Zona “Toluca” del Instituto de Seguridad y Servicios Sociales de los Trabajadores del Estado (ISSSTE), Toluca, MEX; 4 Medicine, Universidad Popular Autónoma del Estado de Puebla, Puebla, MEX

**Keywords:** bilateral thalamic infarction, deep cerebral venous thrombosis, hypercoagulability, magnetic resonance venography, neuroimaging, oral contraceptives, vein of galen, venous stroke

## Abstract

Deep cerebral venous thrombosis (DCVT) represents an uncommon and potentially devastating variant of venous cerebrovascular disease, characterized by the occlusion of the Galenic system and the straight sinus. This pathology disrupts the drainage of critical diencephalic structures, resulting in venous congestion that radiologically manifests as symmetrical bilateral thalamic edema or infarction. We report the case of a 56-year-old female with a history of chronic oral estradiol valerate and levonorgestrel use for hormone replacement therapy, who presented with acute neurological impairment, including deep somnolence and temporospatial disorientation. Laboratory tests revealed elevated D-dimer (2020 ng/ml), and neuroimaging confirmed the absence of flow in the deep venous sinuses with bilateral thalamic involvement. The patient was treated with therapeutic doses of enoxaparin, showing remarkable clinical improvement and regaining full orientation within eight days. DCVT must be considered a primary differential diagnosis in patients with impaired consciousness and symmetrical bilateral thalamic lesions, as early recognition of procoagulant risk factors and immediate anticoagulation are decisive in preventing permanent sequelae.

## Introduction

Cerebral venous thrombosis (CVT) represents a heterogeneous and challenging clinical entity, accounting for approximately 0.5% to 1% of all strokes in the adult population [1]. Within this spectrum, deep CVT (DCVT) is an exceptionally rare variant, accounting for less than 10% of all CVT cases [2]. This condition involves occlusion of vital structures, including the internal cerebral veins, the vein of Galen, and the straight sinus, thereby interrupting drainage from the brain's deepest subcortical structures [3].

The pathophysiology of DCVT is characterized by a sudden increase in capillary and venous pressure that exceeds interstitial oncotic pressure, leading to acute vasogenic edema [4]. This often manifests as a "thalamoscopic syndrome," characterized by impaired consciousness, cognitive disorders, and oculomotor abnormalities, frequently without the classical signs of intracranial hypertension [5,6]. Advanced neuroimaging, specifically magnetic resonance imaging (MRI) and magnetic resonance venography (MRV), remains the gold standard for diagnosis, as it enables characterization of bilateral thalamic involvement [7]. Early identification of procoagulant risk factors, such as hormonal therapy, is crucial for initiating timely anticoagulant treatment [8]. Therefore, neuroimaging plays a crucial role not only in confirming deep venous occlusion but also in ruling out critical arterial mimics of bilateral thalamic lesions, most notably an artery of Percheron infarction.

## Case presentation

A 56-year-old female with a long-term medical history of postmenopausal hormone replacement therapy, utilizing a continuous combined oral formulation of estradiol valerate (2 mg) and levonorgestrel (0.15 mg) once daily for the past five years, was admitted to the emergency department following acute neurological impairment. The clinical presentation was characterized by deep somnolence, bradypsychia, bradylalia, and temporospatial disorientation. Upon neurological examination, the patient exhibited significant memory impairment and altered executive functions; however, cranial nerves were intact, and no focal motor or sensory deficits were noted.

Initial laboratory workup was performed upon admission to evaluate metabolic, inflammatory, and coagulation status. The most significant findings were a markedly elevated D-dimer (2020 ng/mL) and an atherogenic lipid profile, which were considered contributory chronic vascular risk factors rather than direct triggers for venous thrombosis. Comprehensive results, including institutional reference ranges, are summarized in Table 1.

**Table 1:** Clinical laboratory results at admission with institutional reference ranges INR: international normalized ratio, LDL: low-density lipoprotein, HbA1c: glycated hemoglobin, TSH: thyroid-stimulating hormone, HIV: human immunodeficiency virus, HBsAg: hepatitis B surface antigen, anti-HCV: antibodies to hepatitis C virus

Laboratory parameter	Patient result	Reference range	Interpretation
Hematology			
Hemoglobin	15.1 g/dL	13.0-18.0 g/dL	Normal
White blood cell count	13.29 x 103/µL	4.0-12.0 x 103/µL	High
Neutrophils (%)	76.20%	40.0-85.0%	Normal
Platelets	262 x 103/µL	130-450 x 103/µL	Normal
Coagulation and inflammatory			
D-dimer	2020 ng/mL	<500 ng/mL	Critical elevation
Prothrombin time	11.2 sec	11.5-15.1 sec	Slightly low
INR	1.02	0.9-1.4	Normal
Activated partial thromboplastin time	21.7 sec	25.3-38.0 sec	Shortened
Chemistry and metabolic			
Glucose	105 mg/dL	70-99 mg/dL	High
Creatinine	0.9 mg/dL	0.7-1.1 mg/dL	Normal
Total cholesterol	248 mg/dL	<200 mg/dL	High
LDL cholesterol	169 mg/dL	<100 mg/dL	High
Triglycerides	197 mg/dL	<200 mg/dL	Normal (borderline)
HbA1c	5.60%	4.8-5.9%	Normal
Electrolytes			
Sodium	136 mmol/L	136-145 mmol/L	Normal
Potassium	4.0 mmol/L	3.5-5.1 mmol/L	Normal
Thyroid and viral panel			
TSH	3.30 µUI/mL	0.27-4.20 µUI/mL	Normal
HIV 1/2 and Ag p24	Non-reactive	Non-reactive	Normal
Hepatitis B (HBsAg)	Non-reactive	Non-reactive	Normal
Hepatitis C (anti-HCV)	Non-reactive	Non-reactive	Normal

**Figure 1:**
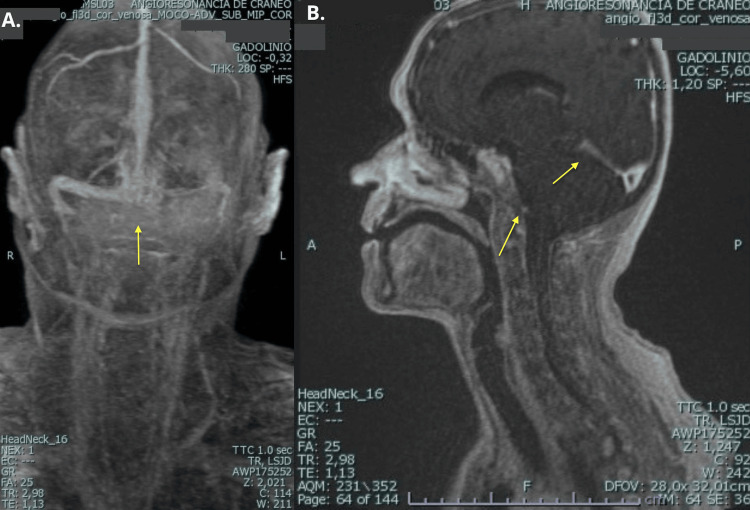
MRI and MRV findings MRA of the head, venous phase (MRV). (A) Coronal MIP reconstruction demonstrating the absence of normal flow and a distinct filling defect within the straight sinus, extending from the deep venous system. (B) Contrast-enhanced (gadolinium-enhanced) T1-weighted sagittal view showing signal alteration and thickening secondary to thrombosis involving the straight sinus, vein of Galen, and internal cerebral veins. MRA: magnetic resonance angiography, MRI: magnetic resonance imaging, MIP: maximum intensity projection

Subsequent MRI and MRV confirmed the diagnosis, showing a complete absence of flow in the deep venous system, specifically involving the internal cerebral veins and the vein of Galen. Symmetrical bilateral thalamic hyperintensities were identified on T2-weighted and fluid-attenuated inversion recovery (FLAIR) sequences, consistent with vasogenic edema secondary to venous congestion (Figure 1).

The patient, who weighed 60 kg, was started on weight-based therapeutic anticoagulation with low-molecular-weight heparin (enoxaparin 60 mg subcutaneously every 12 hours, equivalent to 1 mg/kg) alongside aggressive hyperhydration. An extensive systemic workup, including immunological and rheumatological screening (ANA, anti-DNA, and lupus anticoagulant), was negative. Specialized molecular and functional assays for hereditary primary thrombophilias, including the factor V Leiden mutation, the prothrombin G20210A mutation, and assays for protein C, protein S, and antithrombin III deficiencies, were not performed due to institutional resource limitations during the acute setting and the presence of a clear, transient provoking factor (long-term hormonal therapy). Consequently, the possibility of an underlying genetic predisposition acting synergistically with the oral contraceptives cannot be entirely excluded. Following eight days of inpatient management, the patient showed remarkable clinical recovery, with complete resolution of cognitive deficits and language fluency. Upon discharge, the sequential oral therapies of estradiol valerate and levonorgestrel were permanently discontinued to mitigate the risk of recurrence. The patient was transitioned to oral anticoagulation with a direct oral anticoagulant (rivaroxaban 20 mg once daily) for a planned duration of six months, in accordance with current international guidelines for provoked CVT. A follow-up MRV was scheduled at the three-month post-discharge outpatient clinic to document venous recanalization. At her initial one-month outpatient evaluation, she remained entirely asymptomatic with no residual neurological or cognitive sequelae.

## Discussion

DCVT is a rare and potentially devastating variant of venous cerebrovascular disease, accounting for approximately 10% of all CVT cases [1,2]. Unlike the more common thrombosis of the superficial dural sinuses, DCVT involves the occlusion of the internal cerebral veins, the vein of Galen, and the straight sinus [3,4]. This anatomical involvement leads to a marked increase in venous perfusion pressure, which exceeds interstitial oncotic pressure, resulting in symmetrical congestion and vasogenic edema of the thalami and basal ganglia, a pattern frequently referred to as "thalamoscopic syndrome" [5,6].

In the context of bilateral thalamic lesions, a rigorous differential diagnosis is mandatory to rule out arterial mimics, most notably an artery of Percheron infarction. Artery of Percheron occlusion typically presents with acute neurological impairment and symmetrical thalamic hyperintensities on T2/FLAIR sequences, mimicking the "thalamoscopic syndrome" observed in DCVT. In our patient, an arterial ischemic stroke was conclusively excluded based on two primary findings: first, the lack of acute restricted diffusion strictly to an isolated arterial territory on diffusion-weighted imaging, and second, the definitive demonstration of complete absence of flow within the internal cerebral veins and the straight sinus on the venous phase of the MRI (MRV). Documenting the occlusion of the deep venous drainage pathway confirmed that the pathogenetic mechanism was venous congestion and vasogenic edema rather than primary arterial ischemia [7].

The clinical presentation of DCVT is often non-specific, characterized by fluctuations in consciousness, cognitive impairment, and memory deficits [8]. In this case, the patient’s history of chronic hormonal therapy with estradiol and levonorgestrel was a decisive factor, as synthetic estrogens are well-documented to induce a prothrombotic state [9,10]. In women over 50 years of age, this risk is further amplified, necessitating a thorough evaluation of the deep venous system when symmetrical bilateral thalamic lesions are identified [11,12].

Advanced neuroimaging remains the gold standard for diagnosis. While non-contrast CT may show hyperdensity in the straight sinus, MRI and MRV are essential for confirming the absence of flow and the extent of thrombosis [13]. The presence of definitive filling defects and the loss of the normal flow void within the deep venous system are the primary radiological hallmarks for this condition [14]. Early recognition is vital, as timely initiation of anticoagulation therapy, primarily with low-molecular-weight heparin, is the most effective intervention to reverse venous congestion and prevent permanent neurological damage [15]. As demonstrated in our case, rapid clinical improvement and complete resolution of deficits are possible even with significant initial impairment if the underlying etiology is addressed promptly.

This case report has some limitations that should be acknowledged. First, a comprehensive inherited and acquired thrombophilia panel, including factor V Leiden, prothrombin gene mutations, and protein C/S assays, was not performed due to institutional resource limitations during the acute hospitalization. Although a clear, transient provoking factor (long-term combined oral contraceptives) was documented, an underlying genetic predisposition cannot be completely excluded. Second, while the initial response to anticoagulation was favorable and an outpatient MRV was scheduled for the three-month follow-up, our report is limited by a relatively short clinical follow-up period immediately following discharge. Long-term monitoring is required to definitively document complete venous recanalization and rule out delayed neurological or cognitive sequelae.

## Conclusions

DCVT is a rare clinical entity that must be considered in patients presenting with acute cognitive impairment and symmetrical bilateral thalamic lesions. This case underscores the diagnostic challenge posed by non-specific symptoms and the decisive role of advanced neuroimaging, specifically MRV, in confirming deep venous system occlusion. Furthermore, identifying modifiable risk factors, such as long-term hormonal therapy in postmenopausal women, is crucial for etiological assessment. Early recognition and the timely initiation of therapeutic anticoagulation remain the most significant factors in reversing venous congestion and preventing permanent neurological sequelae, leading to the favorable clinical outcome observed in this patient.
